# An internet-based survey exploring the awareness of febrile neutropenia in patients with malignant lymphoma

**DOI:** 10.1007/s00520-019-05231-z

**Published:** 2020-01-17

**Authors:** Yasushi Takamatsu, Isao Yoshida, Minoru Yoshida

**Affiliations:** 1 Division of Medical Oncology, Hematology and Infectious Diseases, Department of Internal Medicine, Fukuoka University School of Medicine, 7-45-1 Nanakuma, Jonan-ku, Fukuoka, 814-0180 Japan; 2 Department of Hematologic Oncology, National Hospital Organization Shikoku Cancer Center, 160 Kou, Minamiumemoto-machi, Matsuyama, 791-0280 Japan; 3 Fourth Department of Internal Medicine, Teikyo University School of Medicine, Mizonokuchi hospital, 5-1-1 Futago, Takatsu-ku, Kawasaki, 213-8507 Japan

**Keywords:** Malignant lymphoma, Febrile neutropenia, Internet-based survey, Patients’ awareness

## Abstract

**Purpose:**

Febrile neutropenia (FN) is associated with infection-related mortality and a reduction of relative dose intensity during chemotherapy of malignant lymphoma. To prevent deaths and recover the attenuated efficacy of chemotherapies caused by FN, guidelines for the management of FN are published. The aim of this study is to clarify the degree to how much patients understand the FN.

**Methods:**

An internet-based survey was employed to investigate the awareness of FN in Japanese patients who had received chemotherapies for malignant lymphoma.

**Results:**

A total of 105 subjects were enrolled, of whom 64 (61.0%) received ambulatory treatment and 41 (39.0%) received primarily inpatient services. Sixty-four (61.0%) remembered receiving explanations of FN and 61 (95.3%) understood FN very well or almost well. Respondents who reported understanding received explanations from multiple medical staff that were similar to those from single medical staff. A total of 31 (29.5%) experienced FN and 17 of them developed FN at home. Only 8 (47.1%) visited or made contact with their hospitals within 3 h after onset at home.

**Conclusions:**

Explanatory procedures need to be addressed, since subjects’ levels of understanding were not proportionate to the number of elucidators. Although medical professionals made preliminary explanations, in fact, no more than half of those respondents who developed FN at home had made contact to their hospitals immediately. In conclusion, these results suggest that medical professionals should make more effort to lead patients to an understanding of the proper correspondences in case of FN onset.

## Introduction

Malignant lymphoma is a chemosensitive tumor and a potentially curable disease with cytotoxic chemotherapies including CHOP therapy. The most commonly observed and serious adverse event of cytotoxic chemotherapy is myelosuppression, especially neutropenia, which increases the risk of life-threatening bacterial infection. Febrile neutropenia (FN) is recognized as a medical emergency requiring empiric broad-spectrum antibiotics administration immediately, because a prolonged time-to-antibiotic administration sometimes results in fatal outcome [1]. In addition, FN occasionally cause dose reductions and treatment delays in the following cycles of chemotherapy, leading to decreasing relative dose intensities and then impairing the clinical outcomes of underlying malignancy [2]. To prevent FN-related deaths and improve the efficacy of chemotherapies, guidelines for the use of antimicrobial agents in patients with FN were published by the Infectious Diseases Society of America in 1997 [3] and updated in 2010 [4]. We then have developed a Japanese guideline for the management of FN in 2012 [5] and revised it in 2017. This guideline has been widely recognized by medical staff involved in cancer chemotherapies. However, we have not yet verified whether the guide can contribute to a better management of FN in practice.

According to chemotherapies shifting from an inpatient to an outpatient setting, the number of patients developing adverse events, including FN, has been increasing at home. It is therefore important for patients receiving outpatient chemotherapies to understand how much serious the FN is and what to do when they have a fever. Medical staff, including medical doctors, nurses, and pharmacists, certainly gives patients a detailed explanation of FN before starting chemotherapies. However, there is little data on understanding levels of the patients. We therefore conducted the internet-based survey to explore the awareness of FN in Japanese patients who have received chemotherapies for malignant lymphoma.

## Methods

### Internet-based survey

The authors conducted an internet-based study using an Internet survey system operated by Macromill Inc. (Tokyo, Japan). All respondents were registered as the patient panel of Macromill and its alliance partner. Respondents were eligible if they fulfilled all of the following screening criteria: having been diagnosed with malignant lymphoma; aged 20 years or older; having received any anti-lymphoma chemotherapy within the last 5 years; and providing his/her informed consent to participation of the Internet survey.

To confirm respondent eligibility, we conducted a two-step screening process. First, panels registered in the database of Macromill were asked the confirmation test, and those who matched any of the following criteria were excluded: patients with malignant lymphoma who never received chemotherapy; more than 5 years from his/her last chemotherapy treatment; persons with no response, pre-mature interruption, or insufficient contents during the screening. After several days, panels were asked the same questions as a second screening, and those whose responses were not exactly equivalent to precedence answers were excluded.

This internet-based study was conducted for eligible subjects from December 2017 to February 2018. Respondents were asked questions related to the awareness and management of FN in addition to their characteristics, including age, gender, occupation, house environment, time from home to hospital, current therapeutic situation, time from last chemotherapy, and treatment location.

### Data analysis

Subjects’ response data were tabulated and diagrammed (i.e., pie and/or bar chart) for each question. Overall, data were stratified using age, gender, occupation, house environment, and treatment location. To evaluate the relationship between selected questions, cross tabulations were prepared. Because of the limited sample size, especially for sub-groups, statistical analyses (estimations and/or hypothesis tests) were not generally performed.

Subjects’ demographic and clinical characteristics and survey completion data were analyzed descriptively using mean ± SD, medians, and ranges (minimum and maximum) for continuous variables and counts (percentage) for categorical ones.

### Ethics statement

Respondents had only spent his/her time for the study approximately 15 min responding to the questionnaire. The first screen of the internet survey displayed explanations of the study and asked for respondents’ informed consent (IC). Respondents’ IC were officially recognized when the panel clicked the agree button in the screen. Only the person giving the IC was able to browse the following screens and respond to the questions. The questionnaire screen was able to open using subject’s ID and password.

The computer system in Macromill made respondent’s responses automatically anonymous, and only a limited staff in Macromill other than persons involved in the survey was able to make the responses non-anonymous.

Ethical approval of the study was obtained by the Institutional Review Board of Fukuoka University. The implementation of the internet survey, preparation of patient panels, database management, and data analyses were performed by Macromill Carenet Inc. (Tokyo, Japan) and its associated company, Macromill Inc. This study was sponsored by Kyowa Hakko Kirin Co. Ltd. (Tokyo, Japan), but the survey activities were independent from them.

## Results

### Subject’s characteristics

Among 42,125 panels of Macromill and its alliance partners, 263 responded as patient with malignant lymphoma in the primary screening. In the second screening, patients who conflicted with at least one of exclusion criteria such as no experience of anti-lymphoma chemotherapy, more than 5 years past from the last therapy, not consenting to the research, and incomplete responses to questionnaires, as well as whose answers were inconsistent with between first and second screenings, were excluded. As a result, a total of 105 panels were eligible and registered to this study.

The background characteristics of respondents are shown in Table 1. The median age was 56 years (range, 27–80). Age distribution was as follows: there were 28 subjects (26.6%) aged 49 or younger (young adult), 38 (36.2%) aged 50–59 years (middle age), 32 (30.5%) aged 60–69 years (old age), and 7 (6.7%) aged 70 years or older (very old age). There were 70 males (66.7%) and 35 females (33.3%). Sixty-five subjects (61.9%) were employed, 18 (17.1%) were full-time housewives, and 16 (15.2%) were unemployed including retired. In terms of home environment, 98 respondents (93.3%) lived together with their family and 7 (6.7%) lived alone. Time from home to hospital was less than 1 h for 86 subjects (81.9%) and longer than 1 h for 19 (18.1%). Twenty subjects (19.0%) were under treatment at the time of the survey, while 85 (81.0%) had finished their therapies. Sixty-four subjects (61.0%) received chemotherapies mainly in clinics as outpatients, whereas 41 (39.0%) were primarily admitted to hospitals.

**Table 1:** Subject’s characteristics (*n* = 105)

Items	Number	Percentage (%)
Age (years)		
≤ 49	28	26.6
50–59	38	36.2
60–69	32	30.5
≥ 70	7	6.7
Median (range)	56 (27–80)	
Gender		
Male	70	66.7
Female	35	33.3
Occupation		
Employee	65	61.9
Full-time housewife	18	17.1
Unemployed (incl. retirement)	16	15.2
Others	6	5.7
House environment		
Living together	98	93.3
Living alone	7	6.7
Time from home to hospital		
< 1 h	86	81.9
≥ 1 h	19	18.1
Current therapeutic situation		
Under treatment	20	19.0
Follow-up	85	81.0
Time from last chemotherapy		
< 2 years	60	57.1
2–5 years	45	42.9
Treatment location		
Outpatient treatment, mainly	64	61.0
Inpatient treatment, mainly	41	39.0

**Fig. 1:**
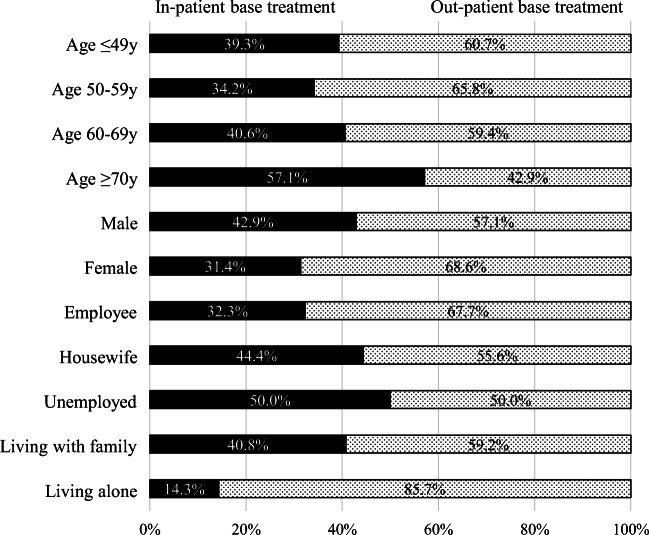
Background profiles of treatment situations by inpatient and outpatient base. Sixty-four subjects (61.0%) received chemotherapies mainly in clinics as outpatient, while 41 (39.0%) were mainly admitted to hospitals. Background profiles by in- and outpatient base were analyzed. Percentage indicates the rates for corresponding sub-group

Since we expected much less respondents who had received chemotherapies upon admission, we analyzed background profiles based upon inpatient and outpatient settings (Fig. 1). The proportions of subjects with inpatient treatment were 39.3%, 34.2%, and 40.6% in younger adult, middle age, and old age segments, respectively, whereas the proportion increased to 57.1% in the very old age segment. A total of 42.9% males and 31.4% females received inpatient treatment. A total of 32.3% of employed respondents, 44.4% of housewives, and 50.0% of those who were unemployed were treated mainly on admission. The proportion of inpatient treatment was 40.8% for subjects living together with family, but only 14.3% for those living alone.

### Subject’s anxieties when notified about malignant lymphoma and its therapies

In the question regarding anxiousness about the therapies, 77 subjects (73.3%) indicated feeling anxious when they had received the notification of malignant lymphoma and its therapies, while 28 subjects (26.7%) did not indicate any anxiety. Those 77 subjects were then asked what they felt anxious about by multiple choice manner. In the 6 items of answers, the most common cause for anxiousness was progression and/or relapse of lymphoma (*n* = 59, 56.2%), followed by development of any adverse drug reactions (*n* = 55, 52.4%), insufficient effectiveness (*n* = 43, 41.0%), medical costs (*n* = 42, 40.0%), influences on daily work and/or living (*n* = 40, 38.1%), and prolongation of the treatment period (*n* = 27, 25.7%).

**Fig. 2:**
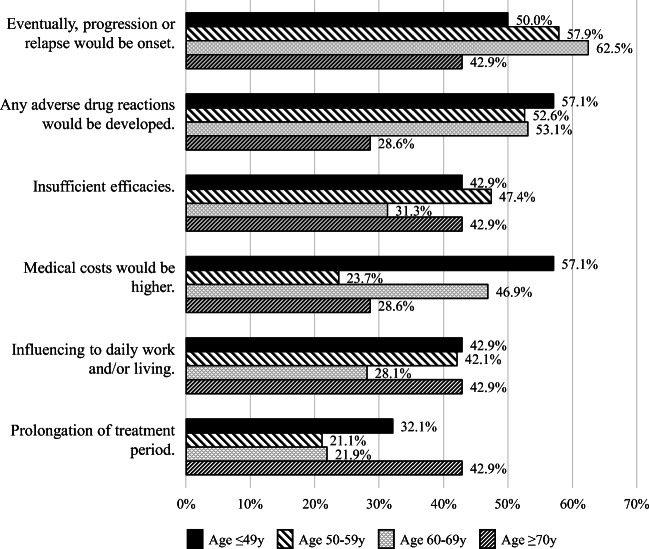
Anxieties on anti-lymphoma therapies for malignant lymphoma. The reasons of the anxious when they had received the notification of malignant lymphoma and its therapies were asked by multiple choice manner. Influences of age on anxiousness were also analyzed. Subjects were classified into a young adult segment (≤ 49 years; *n* = 28), middle age segment (50–59 years; *n* = 38), old age segment (60–69 years; *n* = 32), and very old segment (≥ 70 years; *n* = 7)

The influences of age on selection of items were then analyzed. For middle age and old age segments, the most common anxiety was related to relapse and/or the progression of lymphoma, followed by anxiety about adverse drug reactions, whereas, for young adults, medical costs as well as adverse drug reactions were the highest sources of anxiety (Fig. 2).

In terms of medical costs, the majority of young adults (*n* = 16/28; 57.1%) reported experiencing anxiety, whereas less than half of the middle age (*n* = 9/38; 23.7%) and very old age (*n* = 2/7; 28.6%) segments reported anxiety about this issue. This difference among age segments was the greatest, indicating that the sources of anxiousness were quite different among age segments.

### Explanations of FN

**Fig. 3:**
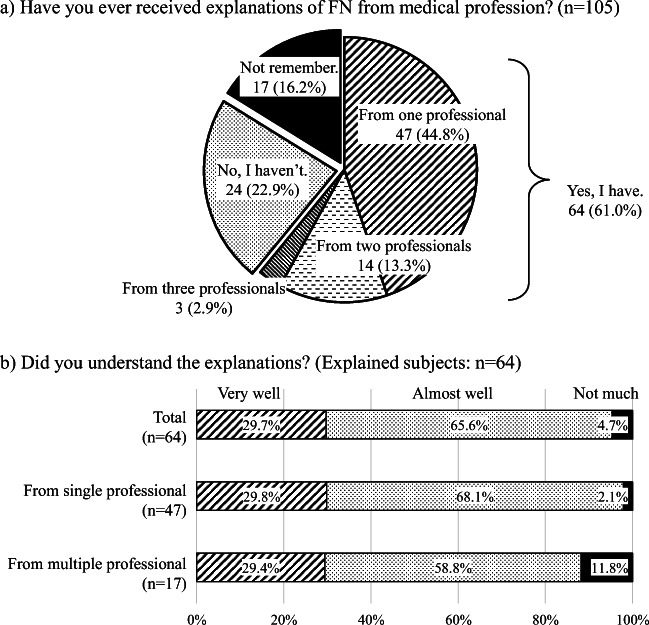
Levels of subjects’ understanding of FN explained by medical professionals. **a** Subjects were asked whether they had received an explanation of FN before starting chemotherapy. **b** The degree of understanding was asked to 64 subjects who answered that they had received an explanation. In these 64 subjects, 47 subjects received an explanation from a single medical staff, while 14 were told by two and 3 by three medical staffs. Levels of subjects’ understanding of FN were compared between single and multiple medical professionals making explanations

Respondents were asked whether they had ever received an explanation of FN prior to starting chemotherapy. Sixty-four subjects (61.0%) replied “yes,” but 24 (22.9%) answered “no” and 17 (16.2%) reported that they “did not remember” (Fig. 3a). The 64 respondents responding “Yes” were also asked about the people who explained FN. A total of 47 subjects received an explanation from a single medical staff, 14 were told by two medical staff members, and 3 were told by three medical staff members.

In terms of their degree of understanding, out of these 64 responding subjects, 19 (29.7%) answered that “I understood it very well,” 42 (65.6%) reported “I almost understood it,” whereas 3 (4.7%) replied “I hardly understood it.”

For the 47 subjects who received an explanation from a single staff, 29.8% and 68.1% of respondents answered “I understood it very well” and “I almost understood it,” respectively, whereas 29.4% and 58.8% of the 17 subjects told by multiple staff members, respectively, reported this response (Fig. 3b). It appears that the level of understanding did not improve regardless of whether the explanation was given by multiple or single medical professionals.

### Development of FN

**Fig. 4:**
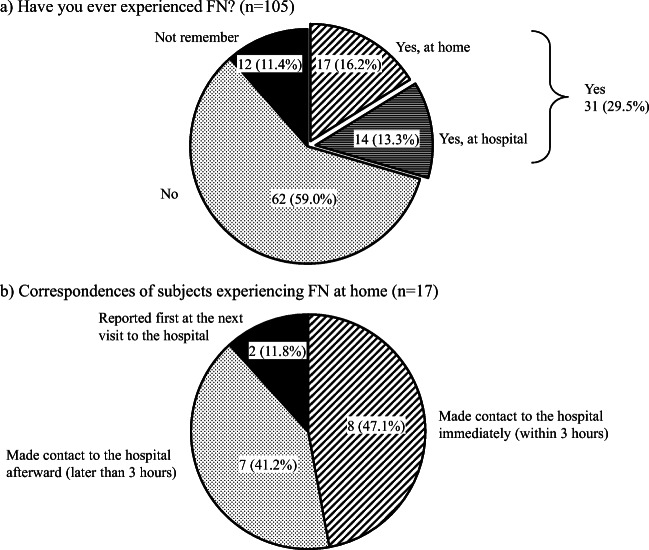
Subjects who had experienced FN. **a** Subjects were asked whether they had ever experienced FN during chemotherapy. **b** A total of 31 subjects had experienced FN, including 17 developing FN at home. These 17 subjects were asked how they had made contact with the hospital

Respondents were then asked whether they had experienced FN during chemotherapy, and a total of 31 subjects (29.5%) answered “Yes” (Fig. 4a). Among them, 17 (16.2%) developed FN at home and 14 (13.3%) at hospital. Meanwhile, 62 (59%) subjects had never experienced FN, and 12 (11.4%) did not remember. The incidences of FN by age were 32.3% in young adult, 18.5% in middle age, 41.9% in old age, and 29.0% in very old age segments. It indicates that there was no apparent difference in the incidences of FN by age in this study.

FNs were developed in 23 (35.9%) of 64 subjects who remembered receiving the explanation of FN, in 3 (12.5%) of 24 who remembered not receiving the explanation, and in 5 (29.4%) of 17 who had vague memories. In 64 subjects who remembered receiving the explanation of FN, FNs were developed in 7 (36.8%) out of 19 subjects who well understood the FN, in 15 (35.7%) out of 42 who almost understood, and in 1 (33%) out of 3 who hardly understood. We investigated the relationship between degrees of FN understanding and incidences of FN, but an apparent one could not be found. The prophylactic effects of G-CSF for FN were uncertain, because the use of G-CSF was not questioned in this study. For the question on severities of FN, 11 subjects (35.5%) replied “I was having a hard time” and 14 (45.2%) “I was having somewhat a hard time.”

### Correspondences of the subjects with FN at home

Out of the 31 subjects with FN, 17 reported developing FN at home. Those 17 subjects were subsequently asked how he/she had made contact for each FN. Eight respondents (47.1%) had visited or made contact with the hospitals immediately (within 3 h). In contrast, 7 respondents (41.2%) had visited or contacted with the hospitals after 3 h. In addition, 2 respondents (11.8%) did not visit or contact with the hospitals until their next scheduled consultation (Fig. 4b).

**Fig. 5:**
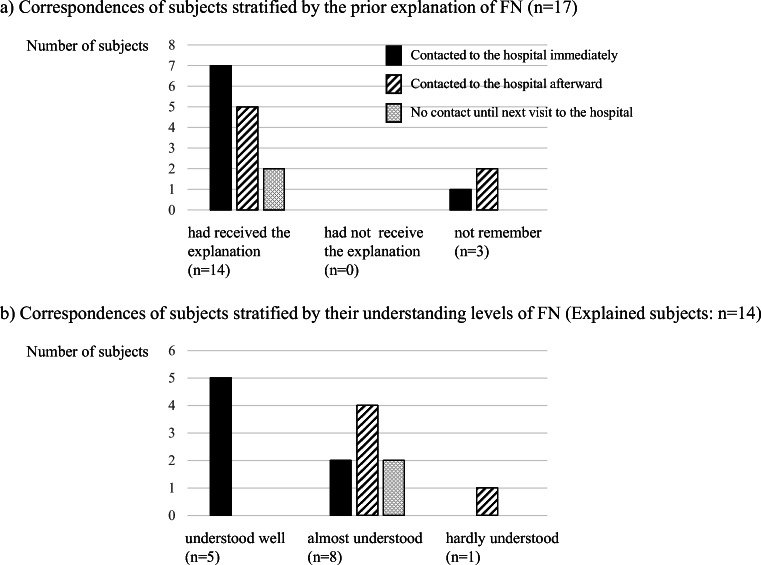
Breakdown of correspondences of subjects experiencing FN at home. **a** A total of 17 subjects developed FN at home. On the 17 subjects, we investigated how much our explanation of FN was reflected to the correspondences of subjects with FN at home. Fourteen subjects out of them remembered receiving the explanation of FN, of 7 (50%) visited or made contact to their hospitals immediately. However, of 5 (35.7%) visited or made contact to their hospitals afterward, and of 2 (14.3%) never visited or made contact to their hospitals until next regular visits. **b** We investigated how much subjects’ understanding levels of FN influenced their correspondences on 14 subjects who remembered receiving the explanation of FN. All of 5 subjects with well understanding surely visited or made contact to the hospitals immediately. However, in 8 subjects with almost well understanding, of only 2 (25%) visited or made contact to the hospitals immediately

On these 17 subjects, we investigated how much our explanations of FN were reflected to the correspondences of subjects with FN at home (Fig. 5a). Fourteen subjects out of them remembered receiving the explanation of FN, of 7 (50.0%) visited or made contact to their hospitals immediately. It shows that, even though subjects had received the explanation of FN, half of them did not make contact to the hospitals immediately. Five (35.7%) of 14 subjects visited or make contact to their hospitals afterward, and 2 (14.3%) never visited or made contact to their hospitals until next regular visits. In residual 3 subjects who had vague memories, one made contact to the hospitals immediately, and other 2 made contact afterward. In subjects who remembered not receiving any explanation of FN, there was no subject developing FN at home.

We next investigated how much subjects’ understanding levels of FN influenced their correspondences on 14 subjects who remembered receiving the explanation of FN and developed FN at home (Fig. 5b). All of 5 subjects with well understanding surely visited or made contact to the hospitals immediately. However, in 8 subjects with almost well understanding, only 2 (25.0%) visited or made contact to the hospitals immediately, and 4 (50%) visited or made contact to the hospitals afterward, but residual 2 (25%) never visited or made contact to the hospitals until next regular visit. In one subject with hardly understanding, he/she visited or made contact to the hospitals afterward. The definitive relationship that was identified as adequate correspondences must require well understanding of FN.

The reasons were questioned to the subjects who did not make immediately contact to the hospitals. There were 2 subjects who never visited or made contact to the hospitals until next regular visit. One of them replied “never instructed to immediately contact to the hospital if I had fever,” and another answered “self-judgement.” The characters of these 2 subjects were as follows: they were male, aged 49 years or younger in one and 60–69 years in another, and both lived less than 1 h from the hospital with their family. Among 7 subjects who visited or made contact to the hospitals afterward, 3 answered “it was at midnight or on a holiday,” 2 replied “never instructed to immediately contact to the hospital if I had fever,” and 2 answered “it might not be problem with such degree of fever.”

## Discussion

Overall, respondents in this study had a median age of 56 years and were predominately male (66.7%). According to the report of National Cancer Center (Tokyo, Japan) in 2018, the age at onset of malignant lymphoma in Japan peaks at 60s–70s, and the ratio of occurrence between males and females is 3:2 [6]. In our study, somewhat younger male patients were registered than other patient populations. It may be that elderly individuals did not register as frequently for the study because it was internet-based, and this population uses the Internet less frequently than other age groups. The number of elderly patients with malignant lymphoma is increasing [6]. Generally, many Japanese elderly patients are living alone or with their aged partner [7], and therefore we divided the subjects into four age segments, i.e., young adult (49 or younger), middle age (50–59 years), old age (60–69 years), and very old age (70 or older) to investigate the influences of age, especially for the older age segment.

A total of 39.0% of subjects received chemotherapies mainly at an inpatient setting. The proportion of respondents primarily treated on admission was higher in those over 70 years of age (57.1%) than younger individuals (34.2–40.6%), and was lower in employed (32.3%), versus unemployed and retirees (50.0%), as expected. However, unexpectedly, more than 30% of respondents younger than 50 years and employees had been treated at an inpatient location. In the clinical practice of CHOP-like therapy, while most of the first cycle of chemotherapies are completed on admission, subsequent cycles are generally administered at an outpatient location. Because, unfortunately, we did not survey subjects’ disease types and therapy regimens in this study; we can only suspect that another treatment regimen, such as EPOCH therapy, which is administered by 96-h-continuous infusion, may have been given [8].

Respondents’ anxieties when initially notified of malignant lymphoma were primarily related to adverse drug reactions and the treatment efficacy, including concerns about relapse and/or progression. Anxieties about medical costs were more frequent in young adult subjects, perhaps because they might earn less incomes and/or have more expenses, such as their child’s educational costs in addition to their medical cost [9]. In contrast, anxiety about medical costs was relatively less in elderly subjects, perhaps because the Japanese healthcare system provides lower self-pay ratios for elderly individuals [10].

A total of 22.9% respondents indicated that they had not received any explanations of FN before starting their chemotherapies. However, it is considered practically impossible to commence any cytotoxic chemotherapies without explanation of FN to patients. We expect that several years can make patients forget the explanation they received, especially for those who had never experienced FN. Additionally, 16.2% of subjects reported not remembering whether they received any explanation or not. On the other hand, even in respondents who had received explanations, 4.7% of them hardly understood. With the above issues taken into consideration, explanations by medical professionals may have insufficiently impressed upon respondents that FN is a serious and significant adverse event, and to help subjects understand what FN is. Our study also reveals that patients’ understanding levels of FN were not improved, regardless of whether single or multiple medical staff members provided explanations to them. One cause of this phenomenon may be that medical doctors, nurses, and pharmacists may conduct explanations independently and repeat very similar contents without confirming the patient’s level of understanding. The method and procedures for explanation needs to involve reconstruction; for example, medical staff makes questions whether he/she has ever received any explanations and to confirm patient’s understanding level of FN.

Based on the results of previously reported clinical trials, the incidences of FN are between 17 and 23% in patients with malignant lymphoma treated by CHOP-like therapies [11–14]. In our study, however, 29.5% of subjects had experienced FN during their treatment. There was no difference in the incidence of FN after adjusting subjects’ age level or degrees of understanding about FN. This finding suggests that the incidence of FN in clinical practice may be higher, perhaps due to including elderly patients and patients with concomitant diseases.

The ASCO guidelines recommend that an empirical antibiotic treatment should be commenced within 1 h of triage [15]. In our study, however, only 47.1% out of subjects who had experienced FN at home visited or made contact to hospitals within 3 h, even though 81.9% of subjects resided within 1 h from hospitals. Surprisingly, another 11.8% of subjects did not visit or make contact with hospitals until their next scheduled consultation. Our findings demonstrated that the better the subjects understood FN, the more rapidly they contacted the hospital when developing FN at home. Since a large majority of subjects had received the explanation of FN but did not recognize what they should do in case of developing FN, it is important that the elucidators themselves or other medical staff should confirm subjects’ current understanding levels of FN. This finding suggests that medical doctors, nurses, and pharmacists should make an effort to improve the patient’s understanding about the most proper correspondence in the case of FN onset.

In conclusion, an internet-based survey was conducted to explore the awareness of FN in Japanese patients with malignant lymphoma who had received chemotherapy. To undergo chemotherapy safely and effectively, it is essential to prevent and manage FN appropriately. However, our study revealed that only 61.0% of subjects had recalled that they had received explanations of FN prior to the commencement of chemotherapy. A total of 16.2% of subjects developed FN at home, and only 47.1% of them immediately visited or made contact with hospitals/clinics. These results suggest that subjects’ level of understanding regarding FN and the proper communication with medical staff are in need of improvement. Physicians and medical staff should recognize these conditions and advance and/or improve the quality of explanations for FN and proper correspondences in practice.

## References

[1] Klastersky J (1993). Febrile neutropenia. Curr Opin Oncol.

[2] Lepage E, Gisselbrecht C, Haioun C, Sebban C, Tilly H, Bosly A, Morel P, Herbrecht R, Reyes F, Coiffier B (1993). Prognostic significance of received relative dose intensity in non-Hodgkin’s lymphoma patients: application to LNH-87 protocol. The GELA. (Groupe d’Etude des Lymphomes de l’Adulte). Ann Oncol.

[3] Hughes WT, Armstrong D, Bodey GP (1997). 1997 guidelines for the use of antimicrobial agents in neutropenic patients with unexplained fever. Infectious Diseases Society of America. Clin Infect Dis.

[4] Freifeld AG, Bow EJ, Sepkowitz KA, Boeckh MJ, Ito JI, Mullen CA, Raad II, Rolston KV, Young JA, Wingard JR, Infectious Diseases Society of America (2011). Clinical practice guideline for the use of antimicrobial agents in neutropenic patients with cancer: 2010 update by the Infectious Diseases Society of America. Clin Infect Dis.

[5] Takamatsu Y (2013). A general description of the clinical guideline for the management of febrile neutropenia. Gan To Kagaku Ryoho.

[6] National Cancer Center Japan Center for Cancer Control and Information Services ganjoho.jp, https://ganjoho.jp/public/cancer/ML/print.htm. Accessed August 2018

[7] Cabinet Office, Government of Japan, annual report on the aging society 2015. (http://www8.cao.go.jp/kourei/english/annualreport/index-wh.html)

[8] Jermann M, Jost LM, Taverna CH (2004). Rituximab–EPOCH, an effective salvage therapy for relapsed, refractory or transformed B-cell lymphomas: results of a phase II study. Ann Oncol.

[9] Statistics Bureau, Ministry of Internal Affairs and Communications, Annual Report on the Family Income and Expenditure Survey (http://www.stat.go.jp/english/data/kakei/index.html)

[10] Ministry of Health, Labour, and Welfare. An Outline of the Japanese Medical System. https://www.mhlw.go.jp/english/policy/health-medical/health-insurance/index.html, Accessed September 2018

[11] Crawford J, Allen J, Armitage J (2011). Myeloid growth factors. J Natl Compr Cancer Netw.

[12] Watanabe T, Tobinai K, Shibata T, Tsukasaki K, Morishima Y, Maseki N, Kinoshita T, Suzuki T, Yamaguchi M, Ando K, Ogura M, Taniwaki M, Uike N, Takeuchi K, Nawano S, Terauchi T, Hotta T (2011). Phase II/III study of R-CHOP-21 versus R-CHOP-14 for untreated indolent B-cell non-Hodgkin’s lymphoma: JCOG 0203 trial. J Clin Oncol.

[13] Lyman GH, Delgado DK (2003). Risk and timing of hospitalization for febrile neutropenia in patients receiving CHOP, CHOP-R, or CNOP chemotherapy for intermediate-grade non-Hodgkin lymphoma. Cancer.

[14] Fisher RI, Gaynor ER, Dahlberg S (1993). Comparison of a standard regimen (CHOP) with three intensive chemotherapy regimens for advanced non-Hodgkin’s lymphoma. N Engl J Med.

[15] Taplitz RA, Kennedy EB, Bow EJ, Crews J, Gleason C, Hawley DK, Langston AA, Nastoupil LJ, Rajotte M, Rolston K, Strasfeld L, Flowers CR (2018). Outpatient management of fever and neutropenia in adults treated for malignancy: American Society of Clinical Oncology and Infectious Diseases Society of America clinical practice guideline update. J Clin Oncol.

